# Associations between body mass index, body composition and bone density in young adults: findings from a southern Brazilian cohort

**DOI:** 10.1186/s12891-019-2656-3

**Published:** 2019-07-09

**Authors:** Isabel Oliveira Bierhals, Juliana dos Santos Vaz, Renata Moraes Bielemann, Christian Loret de Mola, Fernando Celso Barros, Helen Gonçalves, Fernando César Wehrmeister, Maria Cecília Formoso Assunção

**Affiliations:** Postgraduate Program in Epidemiology, Federal University of Pelotas, Rua Marechal Deodoro, 1160 – 3o andar, Pelotas/RS, 96020-220 Brazil

**Keywords:** Cohort studies, Bone density, Nutritional status, Body composition, Obesity

## Abstract

**Background:**

This study aimed to evaluate the association of body composition components and obesity with bone density.

**Methods:**

Prospective study with data on 2968 members of the 1993 Pelotas Birth Cohort from follow-ups at 18 and 22 years of age. Areal bone mineral density (aBMD, g/cm^2^) was evaluated for whole body, lumbar spine, and femoral neck at 22 years using dual-energy X-ray absorptiometry. Simple and multiple linear regression, stratified by sex, were used to assess the effect of BMI, fat mass (FMI) and lean mass index (LMI), evaluated at 18 and 22 years, and obesity trajectories classified by FMI and categorized as “never”, “only at 18 years”, “only at 22 years” or “always” on aBMD.

**Results:**

Among men, the largest coefficients were observed for BMI, followed by lean mass and fat mass. Compared to fat mass, lean mass presented the largest coefficients for all sites, with the strongest associations observed for the femoral neck (β: 0.035 g/cm^2^; 95% CI: 0.031; 0.039 for both follow-ups), while the largest effect for FMI was observed for whole-body aBMD at 18 years (β: 0.019 g/cm^2^; 95% CI: 0.014; 0.024). Among women, the strongest associations were observed for LMI. The largest coefficients for LMI and FMI were observed for femoral neck at age 18, presented β: 0.030 g/cm^2^, 95% CI: 0.026, 0.034 for LMI and β: 0.012 g/cm^2^; 95% CI: 0.009; 0.015) for FMI. Men who were “always obese” according to FMI had smallest aBMD for spine (β: -0.014; 95%CI: − 0.029; − 0.001). Women who were obese “only at 18 years” had smallest aBMD for the whole-body (β: -0.013; 95%CI: − 0.023; − 0.002), whereas those who were obese “only at 22 years” had larger whole-body and femoral neck aBMD (β: 0.013; 95%CI: 0.009; 0.017 and β: 0.027; 95%CI: 0.016; 0.038, respectively) and those “always obese” for whole-body aBMD (β: 0.005; 95%CI: 0.001; 0.011) compared to the reference category.

**Conclusions:**

The indexes were positively associated with aBMD in this sample. Fat mass had smaller positive influence on these outcomes than lean mass, suggesting the most important body composition component for bone density is the lean mass.

## Background

Peak bone mass is reached at the start of adulthood, determines fracture risk in adults [1], and has the potential to delay the onset of advanced age osteoporosis by 13 years [2]. Factors that affect it negatively, particularly during adolescence, can result in an increased risk of fracture and osteoporosis later in life [3].

The interaction between obesity and bone metabolism is complex and has not been entirely elucidated [4]. By 2030, obesity will affect more than one billion people [5–7], and total attributed healthcare costs may reach US$ 957 billion [8]. It had been thought that obesity, when defined as a high body mass index (BMI), had a protective effect on the skeleton [9], since it is related to increased bone mineral content and bone mineral density (BMD) [10–13] and exerts a greater mechanical load on the bones [14]. However, the influence of the two principal components of body weight – fat mass (FM) and lean mass (LM) – on BMD is still a subject of debate [15–18]. While the literature consistently shows that LM has a positive association with bone health [15, 16, 19, 20], the National Osteoporosis Foundation recently concluded that the effect of FM on the accumulation of bone mass in young populations is still open for debate [19].

A wide selection of investigations has observed that adiposity has a negative effect on bone mass [11, 20–24]. In a recent meta-analysis, Dolan et al. [24] stratified samples by age and found that adiposity had a negative effect on the bone mass of people under the age of 25 years, suggesting that the negative influence of increasing adiposity is more striking when bone metabolism is in a state of flux, as is the case during the growth period [24].

The objective of this study was to evaluate the effect of body composition components (FM and LM, evaluated as an index) and BMI at 18 and 22 years and trajectory of obesity among the follow-ups on bone density at 22 years, using data from a population cohort of young adults born in the Southern Brazil and followed since birth.

## Methods

### The 1993 Pelotas birth cohort

In 1993, all maternity units in the city of Pelotas were visited daily, and 5265 births to women residing in the urban area of Pelotas between January 1 and December 31 were identified [25]. A total of 5249 mothers agreed to enroll in the study, and their newborn infants were examined. After the perinatal interviews, subsets were assessed at the ages of 1, 3, and 6 months and at 1, 4, 6 and 9 years. At the ages of 11, 15, 18, and 22 years, all members of the original cohort were invited to further assessments. More detailed information on the methodology employed at follow-up assessments is available elsewhere [25–27].

This study uses data from the follow-ups conducted at 18 and 22 years of age on all cohort members for whom information on body composition and BMI was available from both follow-ups and bone mass from the 22-year follow-up. For the latest follow-up, a digital questionnaire was constructed on the REDCap (Research Electronic Data Capture) [28] platform to enable electronic data collection and subsequent construction of a database.

### Body composition

Body composition variables (FM, LM, and bone mass) were measured using dual-energy X-ray absorptiometry (DXA) (Lunar Prodigy Advance – GE®). These examinations were not conducted with pregnant participants or participants in whom there was a suspicion of pregnancy, wheelchair users, people with bone and joint deformities, or those with weight exceeding 120 kg or height exceeding 192 cm, in accordance with the manufacturer’s instructions. To standardize examinations, participants were given appropriate clothing to wear and did not wear anything made of metal.

Both FM and LM at 18 and 22 years of age were expressed in kilos (kg), using whole body scans, and the respective indices were calculated from the ratio of each variable with the square of weight (kg)/[height(m)]^2^, representing lean mass index (LMI) and fat mass index (FMI), respectively.

Areal bone mineral density (aBMD) (g/cm^2^) was evaluated at 22 years of age for the whole body, lumbar spine (L1-L4), and femoral neck.

### BMI assessment

Weight was measured using a balance connected to an air plethysmography displacement unit (BOD POD® Gold Standard - Body Composition Tracking System) with 10 g precision. Height was measured using a wooden stadiometer with 0.1 cm precision and a maximum amplitude of 2 m. These measurements were taken by examiners who had been trained and standardized using techniques proposed by Habicht [29]. These variables, in both follow-ups (18 and 22 years), were used to calculate BMI from the ratio of body mass to the square of weight (kg)/[height(m)]^2^.

### Obesity classification

Obesity was assessed using FMI classification. In both ages, obesity was classified using cutoffs of 9 and 13 kg/m^2^ for men and women, respectively [30]. Combination of obesity status at both follow-ups was used to classify individuals’ trajectories as “never obese”, “obese only at 18 years”, “obese only at 22 years”, or “always obese”.

### Covariates

The following perinatal variables were investigated as potential confounders: mother’s educational level (0–4, 5–8, 9–11, ≥12 years of study), family income (≤1; 1.1–3; 3.1–6; > 6 times the minimum wage), gestational age (< 34; 34–36; 37–40; > 40 weeks), mother’s pregestational nutritional status (underweight, healthy weight, overweight, or obese), birth weight (< 2500; 2500–2999; 3000–3999; ≥4000 g), and birth length (centimeters). Potential confounders collected at 15 years were self-reported skin color (white; black, brown, or other). Confounders at 18 years were smoking habit (at least one cigarette per day during the month prior to the interview), total physical activity (minutes per week), and daily calcium intake (mg, obtained from a food frequency questionnaire).

### Statistical analysis

All statistical analyses were conducted using Stata 12.1® statistical software (Stata Corp., College Station, Texas, United States) and stratified by sex, since evidence shows that there are sex-linked differences in bone mass [31, 32], and tested for significant interactions (*p* < 0.1). The descriptive analysis used absolute and relative frequencies for categorical variables and means and standard deviations (SDs) or medians and interquartile ranges (p25-p75) for numerical variables. Participants included and excluded were compared using the chi-square test (categorical variables), *t-*test, or Wilcoxon Rank Sum Test (numerical variables), depending on normal or nonnormal distribution of data.

Simple and multiple linear regressions were applied to investigate associations between FMI, LMI and BMI (continuous variables, in kg/m^2^) at each follow-up (at 18 and 22 years of age) and aBMD at 22 years of age. To evaluate the effect according to obesity status at 18 and 22 years on bone mass, simple and multiple linear regressions were also performed, considering “never obese” as a reference category. The association with obesity by FMI was analyzed with an adjustment for LMI. In analyses using continuous exposures, after a test significant (*p* < 0.001) for deviation from linearity between aBMD and BMI for both sexes and for FMI among the men, a quadratic term was included in the respective adjusted regressions.

Beta coefficients, 95% confidence intervals (95% CIs), and *p* values from the Wald test of heterogeneity were calculated to a statistical significance level of 5%. When adjusting for possible confounding factors, variables were included in the regressions according to a complete adjustment model irrespective of the level of significance of the association with the outcome in bivariate analysis.

### Ethics approval

All 1993 Pelotas birth cohort follow-ups were approved by the Research Ethics Committee at the Medical Faculty of the Universidade Federal de Pelotas, and the most recent ethics approval protocol is number 1.250.366. At all stages, participants (or their legal guardians) signed free and informed consent forms.

## Results

### Participants studied

At 18 years of age, 4106 participants were assessed (follow-up rate: 81.3%), while at 22 years, 3810 individuals were interviewed (follow-up rate: 76.3%). Body composition data were available for 2968 of the participants assessed at both follow-ups, of whom 1560 (52.6%) were female. Table 1 shows the differences between the participants included in this study and the remainder of the cohort. For both sexes, the proportion of participants born with weights in the range 3000–3999 g was greater among those included in the study, and so was the proportion of smokers. In contrast, FMI at 18 years and BMI at both follow-ups were both greater among those excluded.

**Table 1:** Characteristics of participants with complete data at both 18th and 22th-year follow-ups compared with those participants with missing data, loss of follow-up or death, stratified by sex

Variables	*Men*			*Women*		
	Mean or median (SD or p25-p75); %			Mean or median (SD or p25-p75); %		
	Participants included	Participants excluded^1^	*p-value*	Participants included	Participants excluded^1^	*p-value*
	*N* = 1408	*N* = 1195		*N* = 1560	*N* = 1085	
*Perinatal*						
Maternal education (years)	*N* = 1406	*N* = 1193	0.183^a^	*N* = 1557	N = 1085	0.003^a^
0–4	25.3	29.2		26.7	32.0	
5–8	47.6	45.3		46.4	45.2	
9–11	18.6	17.5		19.0	14.5	
≥12	8.5	8.0		7.9	8.3	
Family income (MMW)	*N* = 1388	*N* = 1159	0.039^a^	*N* = 1528	*N* = 970	0.272^a^
≤1	17.9	20.6		17.3	20.3	
1.1–3	42.4	42.3		41.6	40.6	
3.1–6	24.4	20.2		24.9	23.5	
>6	15.3	16.9		16.2	15.6	
Gestational age (weeks)	*N* = 1391	N = 1159	0.321^a^	*N* = 1539	*N* = 1050	0.002^a^
<34	1.2	2.0		1.2	2.9	
34–36	6.2	6.4		7.8	6.7	
37–40	75.8	76.1		77.2	79.3	
>40	16.8	15.5		13.8	11.1	
Maternal nutritional status (BMI)	*N* = 1366	*N* = 1155	0.182^a^	*N* = 1530	*N* = 1046	0.237^a^
Low weight	10.4	9.1		8.0	7.8	
Adequate	67.3	69.7		67.6	71.3	
Overweight	17.9	15.7		19.4	16.5	
Obese	4.4	5.5		5.0	4.4	
Birth weight (grams)	*N* = 1407	*N* = 1188	0.017^a^	*N* = 1559	*N* = 1078	< 0.001^a^
<2500	7.5	10.1		10.9	10.6	
2500–2999	22.5	19.2		26.1	33.4	
3000–3999	64.0	63.3		59.2	53.6	
≥4000	6.0	7.4		3.8	2.4	
Length at birth (centimeters)	*N* = 1395	*N* = 1165	0.901^b^	*N* = 1548	*N* = 1054	0.029^b^
	49.1 (2.3)	49.1 (2.6)		48.5 (2.3)	48.3 (2.3)	
*15 years*						
Skin color	*N* = 1345	*N* = 765	0.108^a^	*N* = 1540	*N* = 673	0.597^a^
White	63.0	66.5		63.4	64.6	
Black, brown or other	37.0	33.5		36.6	35.4	
*18 years*						
Smoking habit	N = 1408	*N* = 606	0.015^a^	N = 1560	*N* = 531	0.005^a^
No	86.0	81.7		88.2	83.2	
Yes	14.0	18.3		11.8	16.8	
Total physical activity (min/week)	N = 1406	*N* = 602	0.203^c^	N = 1559	*N* = 528	0.966^c^
	630.0 (300.0 1110.0)	592.5 (260.0; 1140.0)		270.0 (110.0; 600.0)	280.0 (120.0; 600.0)	
Total calcium consumption (mg)	N = 1406	*N* = 587	0.282^c^	N = 1559	*N* = 517	0.656^c^
	674.8 (488.9; 939.4)	670.6 (479.5; 897.8)		638.1 (449.3; 922.1)	660.2 (477.4; 891.4)	
Lean mass index (kg/m^2^)	N = 1408	*N* = 493	0.281^b^	N = 1560	*N* = 390	0.002^b^
	18.0 (1.5)	18.0 (1.7)		14.0 (1.4)	14.3 (1.6)	
Fat mass index (kg/m^2^)	N = 1408	N = 493	0.012^b^	N = 1560	N = 390	< 0.001^b^
	4.1 (2.9)	4.5 (3.5)		8.3 (3.5)	9.0 (3.9)	
Body mass index (kg/m^2^)	N = 1408	*N* = 570	< 0.001^b^	N = 1560	*N* = 449	< 0.001^b^
	23.0 (3.6)	24.2 (5.5)		23.2 (4.4)	24.6 (5.8)	
*22 years*						
Lean mass index (kg/m^2^)	N = 1408	*N* = 133	0.185^b^	N = 1560	*N* = 217	< 0.001^b^
	18.2 (1.7)	18.0 (2.0)		14.3 (1.7)	14.8 (1.9)	
Fat mass index (kg/m^2^)	N = 1408	N = 133	0.571^b^	N = 1560	N = 217	< 0.001^b^
	5.5 (3.4)	5.7 (4.0)		9.8 (4.1)	11.0 (5.0)	
Body mass index (kg/m^2^)	N = 1408	*N* = 279	< 0.001^b^	N = 1560	*N* = 315	< 0.001^b^
	24.7 (4.2)	26.2 (6.7)		25.1 (5.3)	27.3 (7.3)	
Whole body BMD (g/cm^2^)	N = 1408	N = 139	0.041^b^	N = 1560	*N* = 221	0.532^b^
	1.3 (0.1)	1.2 (0.1)		1.2 (0.1)	1.2 (0.1)	
Lumbar spine BMD (g/cm^2^)	N = 1408	*N* = 164	0.542^b^	N = 1560	*N* = 220	0.886^b^
	1.2 (0.1)	1.2 (0.2)		1.2 (0.1)	1.2 (0.1)	
Femoral neck BMD (g/cm^2^)	N = 1408	N = 164	0.187^b^	N = 1560	N = 220	0.355^b^
	1.2 (0.2)	1.2 (0.2)		1.0 (0.1)	1.0 (0.1)	
*Outcome changes (*Δ) at 18–22 years*						
Whole body BMD change (g/cm^2^)	N = 1408	*N* = 12	0.174^c^	N = 1560	N = 10	0.241^c^
	0.04 (0.02; 0.07)	0.04 (0.01; 0.05)		0.02 (0.01; 0.04)	0.04 (0.02; 0.06)	
Lumbar spine BMD change (g/cm^2^)	N = 1406	*N* = 62	0.514^c^	*N* = 1558	*N* = 32	0.980^c^
	0.06 (0.02; 0.10)	0.05 (0.01; 0.09)		0.04 (0.01; 0.07)	0.04 (0.01; 0.07)	
Femoral neck BMD change (g/cm^2^)	N = 1407	*N* = 59	0.486^c^	*N* = 1547	*N* = 43	0.412^c^
	0.01 (−0.05; 0.06)	0.02 (−0.04; 0.07)		0.01 (−0.03; 0.05)	0.02 (−0.02; 0.10)	

*N* Number of observations, *SD* standard deviation, % percentage, *MMW* monthly minimum wages, *BMI* body mass index, *BMD* bone mineral density (g/m^2^)

*Changes in measurement of BMD at 22y – at 18y

^1^Participants excluded from the analyses due to loss of follow-up or missing data;

^a^*P* value refers to Chi-squared heterogeneity test;

^b^*P* value refers to Student’s t-test;

^c^*P* value refers to Wilcoxon Rank Sum Test

The 1993 Pelotas Birth Cohort, Brazil

Among men, there was a higher proportion of excluded individuals with family income at birth ≤1 minimum wage, and whole-body bone mass was greater among those included. Among the women, there was a smaller proportion among those included whose mothers had an educational level of 0–4 years at the time of their birth and a higher proportion of those born at > 40 weeks than among those excluded. Mean birth length was greater among participants included in the study, whereas mean LMI at 18 and 22 years of age and mean FMI at 22 were greater among those excluded.

### Associations between FMI, LMI, BMI and bone mass

**Fig. 1:**
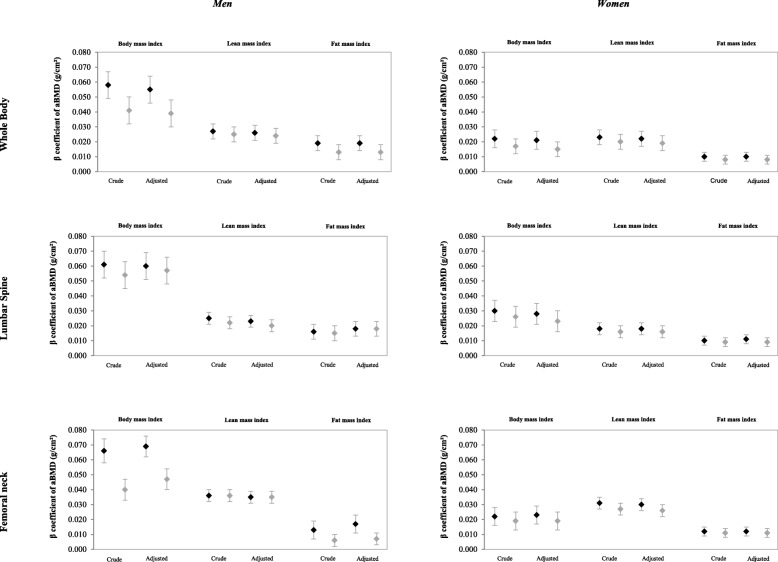
Association between body mass index, fat mass index and lean mass index (kg/m^2^) at 18 and 22 years and bone mineral density (g/cm^2^) at 22 years of age. The 1993 Birth Cohort, Pelotas, Brazil. (*N* = 2968). ♦Black symbols show crude and adjusted coefficients for the follow-up of 18 years and Grey symbols show crude and adjusted coefficients for the follow-up of 22 years of age. Adjusted for perinatal variables (maternal nutritional status, family income, maternal education, gestational age, birth weight, length at birth), 15 years (skin color) and 18 years (smoking habit, total physical activity score, calcium intake)

Figure 1 illustrates the associations between FMI, LMI and BMI at 18 and 22 years and bone mass at 22 years of age. Positive effects of all three indices on bone outcomes were observed and were usually largest for the follow-up at 18 years.

For men, the largest coefficients were observed for BMI, followed by lean mass and fat mass. Compared to fat mass, the lean mass presented the largest coefficients for all sites, with the strongest associations observed for the femoral neck (β: 0.035 g/cm^2^; 95% CI: 0.031; 0.039 for both follow-ups) and whole-body aBMD (β: 0.026 g/cm^2^; 95% CI: 0.021; 0.031 at 18 years and β: 0.024 g/cm^2^; 95% CI: 0.019; 0.029 at 22 years). The largest effect for FMI was observed for whole-body aBMD at 18 years (β: 0.019 g/cm^2^; 95% CI: 0.014; 0.024) and lumbar spine, with the same coefficients for both follow-ups (β: 0.018 g/cm^2^; 95% CI: 0.013; 0.023).

Among women, lean mass presented the largest coefficients of aBMD gain, with the strongest associations for whole-body (β: 0.022 g/cm^2^; 95% CI: 0.017; 0.027 for the 18 years and β: 0.019 g/cm^2^; 95% CI: 0.014; 0.024 for the 22 years) and femoral neck sites (β: 0.030 g/cm^2^, 95% CI: 0.026, 0.034 for age 18 and β: 0.026 g/cm^2^, 95% CI: 0.022, 0.030 for age 22). For FMI, the largest effect was observed at 18 years for all sites, with strongest associations for femoral neck (β: 0.012 g/cm^2^; 95% CI: 0.009; 0.015) followed by lumbar spine (β: 0.011 g/cm^2^; 95% CI: 0.008; 0.014) and whole-body aBMD (β: 0.010 g/cm^2^; 95% CI: 0.007; 0.013).

### Association between obesity and bone mass

Table 2 describes the relationship between obesity, classified by FMI from 18 to 22 years and bone mass at 22 years of age, showing that among men who were “obese” at both follow-ups, there was a reduction in lumbar spine aBMD compared to the reference category (β: − 0.014 g/cm^2^; 95% CI: − 0.029; − 0.001). Among women, those who were obese “only at 18 years” of age presented a reduction in whole-body aBMD (β: − 0.013 g/cm^2^; 95% CI: − 0.023; − 0.002), whereas those who were obese “only at 22 years” and “always obese” presented an increase in whole-body aBMD (β: 0.013 g/cm^2^; 95% CI: 0.009; 0.017 and β: 0.005 g/cm^2^; 95% CI: 0.001; 0.011, respectively). For femoral neck aBMD, women obese “only at 22 years” had a mean increase of 0.027 g/cm^2^ (95% CI: 0.016; 0.038), compared to those “never obese”.

**Table 2:** Association between obesity according to Fat Mass Index (FMI) from 18 to 22 years on bone mineral density (g/cm^2^) at 22 years of age

Variable	*Men (N = 1408)*						
	N	Whole Body		Lumbar Spine		Femoral Neck	
		Mean	Adjusted^a^	Mean	Adjusted^a^	Mean	Adjusted^a^
		(95% CI)	β (95% CI)	(95% CI)	β (95% CI)	(95% CI)	β (95% CI)
Obesity by FMI			0.332		0.140		0.088
Never	1139	1.263	Reference	1.232	Reference	1.169	Reference
		(1.257; 1.268)		(1.224; 1.241)		(1.159; 1.179)	
Only at 18 years	28	1.297	0.008	1.304	0.007	1.188	0.021
		(1.263; 1.331)	(−0.020; 0.005)	(1.251; 1.357)	(−0.016; 0.031)	(1.128; 1.249)	(−0.013; 0.055)
Only at 22 years	158	1.300	0.002	1.249	−0.007	1.204	0.015
		(1.286; 1.313)	(−0.004; 0.008)	(1.228; 1.270)	(−0.017; 0.004)	(1.179; 1.228)	(−0.001; 0.030)
Always	83	1.313	0.005	1.242	−0.014	1.208	0.018
		(1.295; 1.331)	(−0.003; 0.012)	(1.216; 1.268)	(−0.029; − 0.001)	(1.175; 1.241)	(− 0.003; 0.038)
Variable	*Women (N = 1560)*						
	N	Whole Body		Lumbar Spine		Femoral Neck	
		Mean	Adjusted^a^	Mean	Adjusted^a^	Mean	Adjusted^a^
		(95% CI)	β (95% CI)	(95% CI)	β (95% CI)	(95% CI)	β (95% CI)
Obesity by FMI			<0.001		0.339		0.001
Never	1208	1.142	Reference	1.185	Reference	1.007	Reference
		(1.138; 1.146)		(1.178; 1.192)		(1.000; 1.014)	
Only at 18 years	23	1.183	−0.013	1.239	−0.005	1.036	−0.014
		(1.153; 1.212)	(− 0.023; − 0.002)	(1.192; 1.285)	(− 0.027; 0.018)	(0.989; 1.083)	(− 0.042; 0.014)
Only at 22 years	182	1.192	0.013	1.228	0.007	1.080	0.027
		(1.182; 1.202)	(0.009; 0.017)	(1.210; 1.247)	(−0.001; 0.016)	(1.063; 1.097)	(0.016; 0.038)
Always	147	1.236	0.005	1.286	−0.001	1.129	0.006
		(1.225; 1.248)	(0.001; 0.011)	(1.266; 1.305)	(−0.012; 0.010)	(1.109; 1.149)	(−0.008; 0.019)

^a^Adjusted for perinatal variables (maternal nutritional status, family income, maternal education, gestational age, weight and length), 15 years (skin color), 18 years (smoking, total physical activity score, calcium intake) and 22 years of age (lean mass index)

The 1993 Birth Cohort, Pelotas, Brazil

## Discussion

This study investigated the effect of body composition components (FMI and LMI) and BMI at 18 and 22 years and trajectory of obesity on bone density at 22 years. Our results suggest that despite the effect of BMI on bone mass, the impact of lean mass and fat mass differed, with a largest effect observed for lean mass. For both body composition components, the strongest associations were observed at 18 years. According to obesity classification, there was a negative effect in the lumbar spine among men who were “always obese”. For women, the negative effect was observed in the whole body between those who was obese “only at 18 years”. Between those who was obese “only at 22 years” and “always obese” presented a density increase in whole-body and femoral neck.

According to the literature, at age 18, approximately 90% of the bone mass will have been accumulated [33]. The remainder of BMD accumulation occurs during late adolescence, up to the age of 21–25 years. The exact age at which bone accumulation reaches a plateau varies with sex and the region of the skeleton [34]. The peak bone mass of the proximal femur sites occurs around the age of 20 years, while the total body mass reaches its peak between 6 and 10 years later [35]. Many studies have estimated peak bone mass from cross-sectional data [36–38], and others have assessed the longitudinal change [39–42], but only a few have used longitudinal assessment in a population-based sample including teens and young adults [34, 43].

Berger et al. (2010) found that most bone accumulation, especially of the spine and hip, occurs before age 16 in men and women, with more than 94% of peak bone mass already reached by that age [34]. Lu et al. (2016), however, observed that total accumulation ranged from early to late 20s for both sexes, with women reaching their peaks significantly earlier [43]. Additionally, weight, height and BMI had a significant effect on bone tracking [43]. These results indicate that early intervention before and during puberty is necessary to achieve optimal peak bone mass.

The present study confirms that the body composition components affect bone mass with unequal magnitude in an important period of bone accumulation before reaching peak bone mass. This is important because attaining a high peak bone mass in early life predicts a higher bone mass and a reduced risk of osteopenia or osteoporosis later in life [1]. The effects are probably due to different causes, through mechanisms that go beyond the effect of the direct load on the skeleton [15]; genetic, environmental, and hormonal factors are also involved [44–46].

The literature shows that obesity in adulthood can be protective against osteoporotic fractures [9, 10, 18, 47], whereas at younger ages, obesity can have negative effects that are specific to bone [11, 18, 22–24]. Differences in age, severity, and duration of obesity, particularly among longitudinal studies of the subject [48–50], may explain these conflicting results [49–51]. In the current study, the obesity classification revealed a negative association with aBMD in men. Among women, although most of the observed effects were positive, a negative effect was observed among those obese “only at 18 years”. It should be highlighted that this analysis was adjusted for LMI when obesity was classified by FMI, thereby removing the effect of this component. Besides, in both sexes, most of the effects were largest when evaluated at 18 years, showing a lag time between these measures of body composition and bone mass. To confirm this, we performed a transversal analysis to assess the effect of LMI, FMI and BMI at age 18, on bone mass also at 18 years. We can observe that the magnitude of this transversal association was lower, mainly for FMI and BMI exposures (data not shown), reinforcing the existence of this latency period. We also performed analyses on the effect of 18-year exposures on change in bone mass between 18 and 22 years, and we found a positive effect for the whole body, but negative effects could be observed for the sites of the spine and femoral neck.

In addition to mechanical loading, adipose tissue can have an indirect positive effect on bone metabolism via adipokine, cytokines and hormones and can stimulate bone formation by producing estrogens from steroid precursors, increasing the levels of leptin and insulin in the circulation [52–55]. However, adipose tissue also produces adiponectin and cytokines related to inflammation, such as tumor necrosis factor α (TNF-α) and interleukin 6 (IL-6), which can have harmful effects on the bones [52–54, 56]. In the present study, we observed a positive effect of FMI, although it was visibly inferior to that observed for LMI, which leads us to suppose that the small duration of time elapsed between collection of exposure and outcome data may have prevented the manifestation of the negative effect of body fat.

Evidence points to the existence of an FM threshold that, if exceeded during critical periods of skeletal development — particularly in adolescence — may result in skeletal fragility and ultimately a greater risk of fracture [3, 20, 57, 58]. Measures of bone content, density, and strength improve to the extent that LM and FM increase until a “fat threshold” is reached, beyond which additional fat has harmful effects on the growing skeleton [58, 59]. According to a recent meta-analysis, a greater negative correlation between relative adiposity (in percentages) and bone density was observed in obese people (r = − 0.20) than in those who were overweight (r = − 0.08), indicating that the negative impact of adiposity on BMD increases to the extent that adiposity progresses from the overweight category to obese levels, which was particularly evident among men and among those under the age of 25 years [24].

The present study has important strengths, such as aBMD measurements obtained using DXA, the gold standard for bone mass evaluation; a high follow-up rate; the possibility of assessing the association between obesity and bone mass adjusted for potential confounding factors assessed prospectively over the life course, e.g., maternal characteristics at birth and maternal nutritional status; and measurement of exposure at two points in time.

The main limitation of the present study is the short time period investigated. However, in the 1993 cohort, body composition was first evaluated at 18 years of age. We encourage studies of younger cohorts to include the assessment of body composition at early stages to better explore the longer effects of body composition on bone health, including subsequent follow-ups of the 1993 cohort. This recommendation is further justified by the fact that the literature on this topic generally evaluates the relationship between body composition and bone mass in older populations [60–62] and in premenopausal and postmenopausal women [15, 63, 64]. Another limitation is the lack of data on peak bone growth in our population.

## Conclusions

This study observed positive effects of FMI and LMI on bone density at 22 years, with a largest effect observed for lean mass. For both body composition components, the strongest associations were observed at 18 years. According to obesity classification, some negative effects were found at 22 years. These findings emphasize that the body composition components have different effects on bone mass and raise questions about the effects of fat mass at young ages, especially whether the longer time of adiposity exposure may have harmful consequences for bone health.

## Data Availability

The datasets used and/or analyzed during the current study are available from the corresponding author on reasonable request.

## Abbreviations

95% CI: 95% confidence intervals
aBMD: Areal bone mineral density
BMD: Bone mineral density
BMI: Body mass index
DXA: Dual-energy X-ray absorptiometry
FM: Fat mass
FMI: Fat mass index
IL-6: interleukin 6
LM: Lean mass
LMI: Lean mass index
MMW: monthly minimum wages
REDCap: Research Electronic Data Capture
SD: standard deviations
TNF-α: tumor necrosis factor α
WHO: World Health Organization
